# Feasibility of a Web-Based Psychoeducation Course and Experiences of Caregivers Living With a Person With Schizophrenia Spectrum Disorder: Mixed Methods Study

**DOI:** 10.2196/25480

**Published:** 2021-04-23

**Authors:** Anna Laine, Minna Anttila, Heli Hirvonen, Maritta Välimäki

**Affiliations:** 1 Department of Nursing Science University of Turku Turku Finland; 2 Xiangya School of Nursing Central South University Changsha, Hunan China

**Keywords:** caregiver, informal caregiver, internet, mental health, psychoeducation, schizophrenia, mobile phone

## Abstract

**Background:**

Schizophrenia is a severe mental illness that burdens both patients and caregivers.

**Objective:**

The aim of this study is to examine the feasibility of a web-based psychoeducation course targeted at caregivers of persons with schizophrenia spectrum disorders (SSDs) and to describe their experiences of living with a person with SSD based on the material caregivers produced during the web-based course.

**Methods:**

A convergent, parallel, mixed methods study design was used. First, caregivers’ engagement in the course was evaluated quantitatively. Second, the overview of the course feedback was evaluated using quantitative and qualitative methods. Third, the experiences of being a caregiver to a person with SSD were analyzed qualitatively with the thematic analysis of the writings caregivers produced during the web-based course.

**Results:**

A total of 30 caregivers participated in the study and a web-based psychoeducation course. Less than two-thirds (18/30, 60%) completed the course. Content was most often logged for the first module, *Orientation* (3465 log-ins), and the lowest number of log-ins was recorded for the *Daily life* module (1061 log-ins). Feedback on the course varied; over half (10/17, 59%) of the caregivers considered the content to be very good or good, about half (9/17, 53%) considered the website layout to be good, only 6% (1/17) felt that the usability of the website was poor, and no one felt that it was very poor. From the reported experiences of being a caregiver to a person with SSD, 3 themes were formed: the caregiver’s own well-being, relationship with the person with SSD, and experience of health care services.

**Conclusions:**

The web-based psychoeducation course for caregivers living with a person with SSD seems to be especially suitable for those who have little experience as a caregiver. In the future, more planning and the consideration of aspects related to the needs of specific target groups, course content, practical arrangements, and scheduling should be taken into account. In addition, although caregivers can improve their own well-being in different ways, they need regular support and cooperation from health care professionals.

## Introduction

### Background

Schizophrenia spectrum disorders (SSDs) [1] are severe mental health disorders that affect informal caregivers (later caregivers) [2-5]. Over half of the caregivers of patients with schizophrenia experience burden [2,3], often conceptualized as an increased level of stress [6], financial concerns [7], heavy responsibilities [8], and stigmatized experiences [6]. Caregivers also tend to have health problems such as psychological distress [9,10], anxiety [11], or depression [11,12]. In contrast, caring for a person with schizophrenia can also offer positive experiences for family members [13]. Shiraishi and Reilly [13] found in their systematic review of qualitative studies that caregivers may experience family solidarity, admiration, self-confidence, personal growth, and appreciation in their role. These positive impacts on caregivers are individual and related to each caregiver’s coping methods [14]. Mackey and Pakenham [15] also found in their study that coping resources were the most important predictor to better adjustment among caregivers of persons with mental disorders.

Psychoeducation has been found to be effective in reducing relapses and rehospitalization, shortening hospital stays, and increasing adherence in patients with SSD [16]. On the basis of a Cochrane review by Xia et al [16], the results of psychoeducation were better when caregivers were involved. Psychoeducation is recommended as part of treatment in several care guidelines for patients with SSD [17,18]. It can be realized in groups or individually with different kinds of materials, such as written leaflets, videos [16], or web-based materials [19]. During psychoeducation, individuals gain knowledge about the disorder with which they were diagnosed. This increases their understanding of the disorder and helps them to cope with it [16]. In their review of 32 randomized controlled trials with 2858 caregivers, Sin et al [20] found that psychoeducation for caregivers of patients with psychosis reduces caregivers’ global morbidities, negative caregiving experiences, and negative expressed emotions. However, time restraints create an obstacle in face-to-face psychoeducation, as health care professionals need to integrate psychoeducation with other duties [21].

The increased use of digital technologies in health care can strengthen the accessibility of health services [22], and digital technologies offer new opportunities for web-based psychoeducation. A recent review of 46 studies [23] found good acceptance of web-based interventions targeted at caregivers of persons with mental health disorders, neurological disorders, or brain injuries. Web-based psychoeducation interventions have been developed for various caregiver groups, including those with a family member with SSD [24-28]. Web-based psychoeducation has several advantages over face-to-face interventions. Access to web-based psychoeducation is easier because participants can join from home, for example. Web-based psychoeducation also has lower costs [29,30]. The possibility of returning to web-based information when needed can support learning [30], and anonymous participation may reduce stigma [29]. Although access to web-based psychoeducation is easy, it requires an internet connection and a digital device, such as a computer or smartphone. In web-based psychoeducation, participants cannot receive the same level of support from health care professionals as they do with face-to-face interventions, and they must take more direct responsibility for their work. Security issues, such as personal information being shared on the internet, can raise ethical concerns [29].

In previous literature, a variety of web-based psychoeducation programs have been found to have a positive impact on caregivers. Rotondi et al [27] targeted their programs at caregivers and patients with SSD. The program included reading materials with information related to schizophrenia, 3 therapy groups (1 for patients, 1 for caregivers, and 1 for patients and caregivers), the possibility of asking questions from professionals, a library of previously asked questions with their answers, and news and activities in the community. Patients and caregivers used the program independently; however, the sessions were run by a therapist and moderated in therapy forums. During a 14-month period, caregivers used the program for an average of 14 hours, whereas patients used it for an average of 46 hours. Furthermore, another study by Rotondi et al [26] found that the use of the program decreased significantly after the first month. Chan et al [24] described a program that included information related to psychosis in reading materials and videos, information about local mental health services, local and international news, and an interactive discussion forum. In their study, caregivers were satisfied with the program; they visited the website 2-5 times for 5-30 minutes each time. Caregivers reported that the website increased their knowledge about psychosis (85%) and their understanding of local resources (78%). In addition, 84% of caregivers agreed or strongly agreed that they would recommend the website to others. Sin et al [25] studied an intervention for caregivers that included 12 sections. One section was a home page with an introduction and instructions for navigation. Information related to psychosis and caring was divided into 8 sections. Of the 8 sections, 2 sections were for web-based forums, 1 was for experts, and 1 was for peer discussion. In their study, 13 out of 14 caregivers would definitely (72%) or probably (21%) recommend the program to others.

### Objectives

Despite the wide range of existing literature on psychoeducation courses, more research is needed to identify optimal ways of using web-based interventions to support caregivers of people with mental illness [31] and to find solutions to enable their psychoeducation. To our knowledge, research combining a web-based psychoeducation course and the use of its content to evaluate caregivers’ experiences, especially those with a family member with SSD, is sparse [20]. Therefore, in this study, we examined the feasibility of the web-based course targeted at caregivers and described their experiences of being a caregiver for a person with SSD, based on the web-based material caregivers produced during the course. First, we assessed caregivers’ engagement in the web-based course. Second, we collected caregivers’ feedback of the web-based course. Third, we investigated the experiences of being a caregiver to a person with SSD, portrayed in writings produced during the web-based course.

In this study, we use the term *SSD* to refer to *schizophrenia spectrum disorder* based on the 10th version of International Classification of Disorders (ICD-10) categorization F20-29 [1]. The term *caregiver* refers to a patient’s informal caregiver, such as a family member, close friend, or another person close to the patient who takes care of the patient in their daily life [32].

## Methods

### Design

A convergent, parallel mixed methods study design [33] was used to evaluate the use of a web-based psychoeducation course. Both quantitative and qualitative data collection and analysis methods were used to provide a broad view of the topic [34]. First, to evaluate engagement, quantitative methods were used to collect and analyze caregivers’ log records from the web-based psychoeducation course. Second, a combination of quantitative and qualitative methods was used to collect and evaluate caregivers’ numeral and written feedback about the course. Third, qualitative methods were used to collect data portrayed in writings and explore experiences of caregivers caring for a person with SSD. The convergent, parallel design [33] was appropriate, as data were collected at different points in time throughout the course. The study was reported according to Good Reporting of a Mixed Methods Study [35].

### Setting

The study was conducted within FinFami (Finnish Central Association of Families With People With Mental Illness), which is a central organization of 18 local associations based throughout Finland. The target group of the organization is caregivers of people who are recovering from mental illness. The mission is to promote well-being by providing information, support, and hope. With its local associations, FinFami organizes group activities, courses, and events for caregivers [36]. For convenience purposes, 5 local family associations in Southern Finland were invited to participate in the study.

### Eligibility Criteria

Participants were eligible to participate in the study if they met the following inclusion criteria: a caregiver of a person with SSD (ICD-10, F20-F29 [1]), were closely related (eg, parent, spouse, or child) to a person with SSD, were aged 18 years or more, could read and write in Finnish, and were willing to participate in the study. Caregivers were excluded if they were not able to read and write in Finnish, were unable to give informed consent, or the mental illness of their relative was something other than SSD.

### Ethical Issues

This study was approved by the Ethics Committee of the Hospital District of Southwest Finland (ETMK 56/2015). Research permission for data collection was granted by each local family association participating in this study. Consent was not requested from persons with SSD as recruitment occurred in caregiver associations.

During the course, tutors of the course contacted caregivers via phone if there was any concern over the health of the caregiver. Phone calls were also made if topics were too confidential to talk about in the discussion forum or too sensitive to address via email. Tutors were ethically responsible [37] for identifying if a caregiver was in need of professional help, and if they were, the tutors would validate the caregiver’s situation, support them in getting help, and provide more information on the services available for caregivers.

### Sampling and Recruitment

The consecutive sampling method was used to minimize sampling bias by reaching all potential participants during the recruitment phase [38]. First, the researchers approached a volunteer contact person in each local family association. Second, an information session was held for the contact persons to introduce the study and the practical arrangements of the data collection. Third, contact persons were informed about the study in each local association, and caregivers were tentatively invited to participate if they met the inclusion criteria. A total of 3 information meetings for potentially interested participants were arranged for caregivers, during which a short introduction of the study was conducted. The caregivers had an opportunity to introduce themselves and their role as a caregiver. Caregivers were informed about the study aim, methods, possible advantages and disadvantages, ethical issues (voluntariness, privacy, and confidentiality), and practical arrangements [39] of the web-based psychoeducation course and the use of its content to evaluate caregivers’ experiences. Caregivers were also informed in oral and written formats about their right to refuse or withdraw at any phase of the study [39,40].

Caregivers who decided to participate in the study signed an informed consent form after an information session. If they needed more time to think about their participation, they took the consent material home and returned it via post. If a caregiver was willing to participate in the study but was not able to join the information session, written information about the study and a consent form for them to sign was sent to them by post, along with a stamped envelope. Caregivers were able to register for the course after providing consent.

### Web-Based Psychoeducation Course

The web-based psychoeducation course was designed to offer information and peer support to caregivers of persons with SSD. The web-based method was selected because the internet is already used for seeking help and information related to health problems [41], and there is a need to increase the use of web-based methods in health care [22]. The course consisted of papers and written tasks on the Moodle learning platform and an information package on MentalNet, which is a psychoeducation website providing information related to SSD (eg, etiology, symptoms, treatment, daily living, and patients’ rights; ICD-10 codes F20-F29 [1]). The content of MentalNet is based on a literature review; it was designed in cooperation with health care professionals, patients, and caregivers and originally aimed to support patients’ self-management [42]. Nurses have recommended MentalNet as useful for family members, making the website a suitable structure for the course [43]. Anonymity was secured with personal user accounts and passwords on the learning platform. More detailed information about MentalNet is available in other publications [42,43].

The web-based psychoeducation course was hosted by the University of Turku. The length of the course was 8 weeks, and it comprised 6 modules consisting of 6 themes: (1) orientation, (2) mental illness, (3) treatment, (4) daily life, (5) well-being, and (6) patient and caregiver rights. Each module lasted 1 week, except *Orientation*, which lasted 2 weeks. Finally, there were concluding remarks and the opportunity to provide feedback. The deadlines of tasks assigned to caregivers were flexible in case the caregivers needed more time to complete the tasks (Table 1).

**Table 1:** Description of the structure and content of the web-based psychoeducation course.

Module and length	Content	Task of the week
Module 1: Orientation; 2 weeks	Welcome Description of the course Schedule of the course Introduction of course leaders and tutors Service promise News Information and instructions about the learning platform Security and net etiquette Discussion forum	Please read the orientation material and write your expectations from the course. Introduce yourself shortly and anonymously. Describe the well-being of the person you care for and of yourself by answering the following questions: (1) How does the person you care for act, what do they think, and what do they “look like” when they are in poor condition? (2) What does the person you care for look like when they are doing well? (3) What should the person you care for avoid to maintain a good state of mind and a sense of hope? (4) How can you help make this happen? (5) What do you do to maintain your positive state of mind and a sense of hope? (6) What things should you avoid to maintain a positive state of mind and a sense of hope? Voluntary written task: Draw a line on the paper to mark the most important turning points in your life (joys and sorrows). Describe and write what you marked on the paper.
Module 2: Mental illness; 1 week	Information about the origin of SSDs^a^ and its symptoms Additional information about how caregivers can help themselves and their relatives with the illness in different stages of the disorder	Please read the theme “Mental illness” and answer the following questions in writing: (1) What kind of difficult situations have you experienced related to your relative’s disorder? (2) What things have not worked well in getting through difficult situations? (3) What things have worked well in difficult situations that you would like to share with other caregivers? (4) Please comment on other caregivers’ writings in this module.
Module 3: Treatment of the illness; 1 week	Information about the content of care and rehabilitation for SSD Additional information about research concerning the care of SSD and different types of care and restrictive treatment methods	Please read the theme “Treatment” and answer the following questions in writing: (1) What is the meaning of a written treatment plan for your relative? (2) What does it mean for your relative if they are treated in inpatient or outpatient care? (3) What does it mean for you if your relative is treated in inpatient or outpatient care?
Module 4: Daily life; 1 week	Information about support from caregivers Information about different types of support and benefits that might help in managing daily life	Please read the theme “Daily life” and answer the following questions in writing: (1) What kind of things support your relative’s daily life? (2) What kind of things support your own daily life? (3) In what kind of situations is there an increased risk that your relative would not take their medication? (4) What kind of things support your relative in taking their medicine properly?
Module 5: Well-being; 1 week	Information about mental and physical well-being in relationships	Please read the theme “Well-being,” do a writing, and answer and share the following questions in writing with other caregivers: (1) How can you affect your own well-being? (2) How can you decrease the burden caused by the care of your relative?
Module 6: Patient and caregiver rights; 1 week	Information about patients’ rights for sufficient and necessary treatment, self-determination, and their rights to be informed about their treatment and to keep their information confidential Information about compulsory treatment Information about caregivers’ rights in a relative’s treatment	Please read the theme “Patient and caregiver rights” and answer the following questions in writing: (1) How have you acted in situations where a relative with the disorder has not allowed you to take part in their treatment even if you are worried and want to take part? (2) What is good or bad about dealing with health care in your opinion? (3) How would you change practices related to compulsory treatment?

^a^SSD: schizophrenia spectrum disorder.

The caregivers participated in the course at home on their own computers using pseudonyms. For psychoeducation to be truly empowering, it should provide versatile information covering a wide variety of topics (eg, financial help and ethical aspects) in addition to subjects related to the disorder itself [44]. The themes and tasks in the course were structured to provide caregivers with a comprehensive program that would help them identify any problems in their daily lives and explore solutions to the problems. The layout of the course was predesigned [42,43]. However, each participant could tailor the specific content of the 6 modules based on their own needs. Each participant reflected on their own situation and sought answers to their questions using the web-based material available on the course website. The courses were designed to support caregivers’ self-management and well-being and help them recognize their own resources. In addition, caregivers were encouraged to share their thoughts in a discussion forum using a peer support approach. The specific content of the course material in each module was based on a variety of evidence-based sources on the MentalNet website [45,46].

A total of 3 trained professional tutors specialized in mental health provided feedback after each completed module and answered any emerging questions. Of the 3 trained professional tutors, 2 were psychiatric nurses—one was a registered nurse, a family psychotherapist, and a master’s student in nursing science and the other was a doctoral student (a registered nurse with extensive experience in psychiatric nursing). The third tutor had a PhD. Via emails or text messages, the tutors encouraged caregivers to continue in the course, share their worries, and use the psychoeducation website for support. Moreover, a course coordinator answered practical questions related to the study or course and informed the caregivers about the phases of the course.

### Data Collection

#### Summary of Data Collection

Data were collected between November 2015 and January 2016. Sociodemographic information was obtained with a paper questionnaire, either filled out at the end of the information session or returned by post. The quantitative data related to caregivers’ engagement in the web-based course, which was based on the course’s access log, were collected during the course. Quantitative and qualitative feedback of the course was collected after the course (by January 3, 2016) using a web-based questionnaire as part of the course tasks. Finally, caregivers’ experiences were gathered qualitatively from the learning platform after the course. The researcher copied all caregivers’ writings into one Microsoft Word document, which comprised 156 pages (font: Arial 11; spacing: 1.5).

#### Data Collection Methods

##### Feasibility of the Web-Based Psychoeducation Course

###### Engagement in the Course

Caregivers’ engagement in the course was assessed by calculating their activity on the learning platform. Caregivers’ log-ins for each module were automatically recorded on the learning platform. The number of caregivers visiting every module and the number of finalized module tasks (writings) were calculated manually.

###### Feedback on the Course

A questionnaire designed by the research group was used for feedback. The questionnaire included three 5-point Likert scale questions (eg, “My feedback about the content of the psychoeducation course”; 1=very poor; 2=poor; 3=neither good nor poor; 4=good; 5=very good) with items about content, layout, and usability of the course [47,48]. The participants were also invited to provide written feedback about the course and the website.

##### Caregivers’ Experiences of Caring for a Person With SSD

The qualitative data concerning experiences of being a caregiver for a person with SSD were portrayed in writings [49] designed primarily for the psychoeducation course. Caregivers were instructed to read specific course material assigned for each week, complete writing tasks based on their experiences and guided with open-ended questions related to each theme, and return their tasks via the web-based platform (Table 1).

#### Sociodemographic Information

Caregivers were asked to provide background information on a form regarding their age, gender, marital status, educational level, employment status, housing situation, and relationship with the person with SSD. They were also asked to provide some information about the person with SSD (the duration of mental illness and duration of using mental health services). In addition, caregivers were asked about any possible chronic illnesses they might have and their skills and attitudes regarding computers and the internet.

#### Data Analysis

An evaluation of the use of the course was based on an analysis of data collected at different time points. Caregivers’ engagement in the course was evaluated using descriptive analysis methods. The frequencies of the log-ins to each module were calculated. The number of caregivers visiting each module and completing the tasks over the duration of the course was calculated. Participants’ feedback on the course was then evaluated using descriptive statistics (frequencies and percentages).

For written feedback, data were categorized and analyzed using qualitative content analysis [50]. The written feedback was collected into one document and read through so that the researchers could familiarize themselves with the data. Similarities were identified and classified into categories.

To investigate caregivers’ experiences of being a caregiver to a person with SSD, the writings produced during the course were analyzed using thematic analysis. Thematic analysis was chosen because it can be used to identify, analyze, organize, describe, and report themes found in the qualitative data [51]. First, the first author (AL) read all writings several times to become familiar with the data and to get an idea of the experiences of these caregivers. Second, initial codes such as *individual hobby* and *social relations* were generated. During coding, sections of the text were marked, indicating the code or subject. Third, potential subthemes were generated inductively from the initial codes. The marked sections were copied and collected into separate files. Fourth, the coded data and potential subthemes were reviewed and refined by collapsing and separating them to ensure that they were in a coherent pattern. An example of a coding tree is presented in Table 2. The data were reread to confirm that the themes were related to the data set and that nothing had been overlooked. Fifth, the themes were defined and named after identifying what each theme contained and considering an appropriate name [51]. One researcher (AL) conducted the coding process. Two researchers (AL and MA) defined the subthemes and themes.

**Table 2:** Example from a coding tree of caregivers’ well-being.

Phrase in the text	Initial code	Subtheme	Theme
Reading a book	Individual hobby	Hobbies	Caregivers’ experiences of factors supporting well-being
Jogging	Individual hobby	Hobbies	Caregivers’ experiences of factors supporting well-being
Friends	Social relations	Support from other people	Caregivers’ experiences of factors supporting well-being
Family	Social relations	Support from other people	Caregivers’ experiences of factors supporting well-being

## Results

### Participants

**Figure 1:**
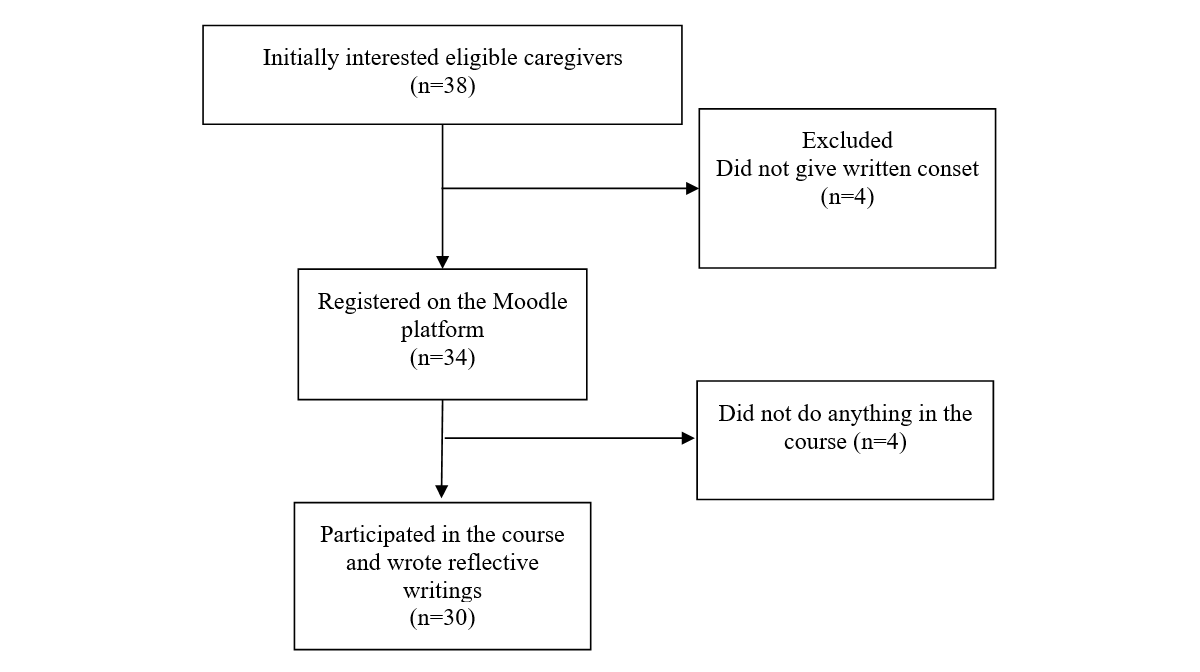
Flow diagram of the participants.

The participant flow and the sociodemographic information about participants are presented in Figure 1 and Table 3, respectively.

**Table 3:** Sociodemographic information of participating caregivers (N=30)^a^.

Characteristic		Value
Age (years; n=29), mean (SD)		59.7 (9.9)
**Gender (n=28), n (%)**		
	Female	23 (82)
	Male	5 (18)
**Marital status (n=28), n (%)**		
	Unmarried	3 (11)
	In common-law marriage or married	17 (61)
	Divorced	6 (21)
	Widow	2 (7)
**Educational level (n=27), n (%)**		
	Primary school	5 (19)
	Vocational school or vocational courses	10 (37)
	Bachelor’s degree	2 (7)
	Master’s degree	10 (37)
**Employment status (n=29), n (%)**		
	Unemployed	2 (7)
	Student	1 (3)
	Retired	16 (55)
	Employed or on sick leave	10 (34)
**Housing situation (n=29), n (%)**		
	Living with a spouse or family	20 (69)
	Living alone	9 (31)
**Relationship with the person with SSD^b^ (n=29), n (%)**		
	Spouse	5 (17)
	Child	6 (21)
	Parent	15 (52)
	Other	3 (10)
**Duration of the illness of the person with SSD (years; n=28), n (%)**		
	0-10	14 (50)
	11-20	11 (39)
	21-30	3 (11)
**Duration of using mental health services (years; n=27), n (%)**		
	0-10	10 (37)
	11-20	14 (52)
	21-30	3 (11)
**Chronic illness of a caregiver (n=29),** **n (%)**		
	No	18 (62)
	Yes	11 (38)
**Computer or internet skills (n=29), n (%)**		
	Very good	7 (24)
	Good	13 (45)
	Neutral	8 (28)
	Quite poor	1 (3)
	Poor	0 (0)
**Attitudes toward computers or internet (n=29), n (%)**		
	Very positive	12 (41)
	Positive	15 (52)
	Neutral	2 (7)
	Negative	0 (0)
	Very negative	0 (0)

^a^The number of responses varied depending on how many participants answered each question.

^b^SSD: schizophrenia spectrum disorder.

### Feasibility of the Web-Based Course

Regarding engagement in the web-based psychoeducation course, the highest number of log-ins was recorded for the *Orientation* module (3465 log-ins), and the lowest number of log-ins was recorded for module 6, *Daily life* (1061 log-ins; Multimedia Appendix 1). A total of 30 (100%) caregivers visited the modules, 25 (83%) caregivers completed at least one of the main modules besides *Orientation*, and 18 (60%) caregivers completed all the 6 modules. The numbers of caregivers who visited and completed the modules are presented in more detail in Multimedia Appendix 2.

### Feedback on the Web-Based Psychoeducation Course

#### Feedback Characteristics

More than half (10/17, 59%) of the caregivers evaluated the content of the psychoeducation as very good or good, about half (9/17, 53%) evaluated the website layout to be good, only 6% (1/17) considered the usability of the website to be poor, and no one felt it was very poor (Table 4).

**Table 4:** Feedback about content, layout, and usability (n=17).

Dimension of the feedback			Caregiver, n (%)
**Content of the website**			
	Very good	2 (12)	
	Good	8 (47)	
	Neither good nor poor	7 (41)	
	Poor	0 (0)	
	Very poor	0 (0)	
**Layout of the website**			
	Very good	0 (0)	
	Good	9 (53)	
	Neither good nor poor	6 (35)	
	Poor	2 (12)	
	Very poor	0 (0)	
**Usability of the website in psychoeducation**			
	Very good	2 (12)	
	Good	5 (29)	
	Neither good nor poor	9 (53)	
	Poor	1 (6)	
	Very poor	0 (0)	

Caregivers’ written feedback about the course was divided into 2 categories: (1) content of the course and (2) course arrangements.

#### Content of the Course

The caregivers found the content of the course to be interesting. The theoretical information was considered good, and caregivers used it in conversations with professionals. In addition, the tasks were found to be good. Caregivers found the writings of other participants valuable because they could compare their own situation with others’ situation and read how others had survived. However, they were of the opinion that the content of the course did not provide new information for long-time caregivers. They did not find peer support when there were no similar situations or there was only limited conversation between the caregivers. In addition, they found it depressing to read others’ stories.

#### Course Arrangements

The caregivers found that the course was well constructed. Caregivers learned how to use the learning platform during the course. There were, however, some technical problems, especially at the beginning of the course. The caregivers felt that the end of the year was not a good time to take part in the course. They found that there was too little time allocated for each module. In addition, the information meeting at the beginning could have been a web-based conversation instead of a face-to-face meeting.

### Experiences of Caregivers Caring for Persons With SSD

#### Themes and Subthemes

An overview of the themes and subthemes is presented in Textbox 1.

**Themes and subthemes.**

**Influence on caregivers’ lives**

- Experiences of the impact of illness on the caregivers themselves
  Physical well-being
  Mental well-being
- Factors supporting the caregivers themselves
  Positive mood
  Hobbies
  Everyday routines
  Support from other people and the health care system

**Relationship with the person with schizophrenia spectrum disorder**

- Caregivers’ experiences of a good relationship
  Help in everyday life
  Mental support
  Help related to the illness
- Caregivers’ experiences of challenges in a relationship
  Physical distance
  Challenges related to schedules
  Difficulties in communication
  Challenges related to stage of the illness

**Experience of health care services**

- Caregivers’ positive experiences in the treatment of the illness
  A sense of security created by hospital care
  Benefits of outpatient care
  Good cooperation with health care
- Challenges faced by caregivers in the treatment of the illness
  Inadequacy of outpatient care
  Challenges of multiple diagnosis
  Challenges in cooperation between caregivers and health care
  Challenges related to commitment in care
  Challenges related to legislation

#### Influence on Caregivers’ Lives

The influence on caregivers’ lives was divided into 2 themes: (1) experiences of the impact of illness on the caregivers themselves and (2) factors supporting the caregivers themselves. Living with a person with SSD has an impact on caregivers themselves. Caregivers expressed signs of depression with feelings of sadness, tiredness, and dejection. Burdening feelings appeared when caregivers were worried about the management of the person with SSD and other family members. They also described a link between the well-being of the caregiver and that of a person with SSD. For example, caregivers found that their own well-being worsened when the state of illness of the person they cared for deteriorated:

After all, the camel’s back was broken because I was and lived alone. In addition to physical disorders, depression hit as it hits many other caregivers.

During the illness, the fear of mine has been related to coping of other family members.

I notice that my own mood depends on my son’s situation.

Caregivers described events or occasions that supported them. These included positive mood, everyday routines, hobbies, and support from other people. Caregivers tried to keep themselves positive by using humor to get through hard moments and tried to avoid energy-consuming people and events. Maintaining regular routines, such as maintaining proper nutrition and regular rest, helped to manage daily activities. Hobbies, including exercise, cultural activities, societal activities, voluntary work, and traveling, gave meaning to their lives. Caregivers received support from other people, such as family members and friends, who helped in difficult times. Colleagues also showed support, as they helped caregivers focus on work. Support from the official social and health service sector included discussions with professionals when the relative was in hospital. Support from the service sector consisted of peer support and family association meetings. Caregivers understood that the passage of time supported their well-being and that they needed to take care of themselves to support others and live their own life:

Focusing on “normal things,” sense of humour and friends are what carries me if it otherwise goes against the walls.

I try to do things I like: I meet people, I have hobbies like studying and exercise, theatre and travelling.

When I feel particularly difficult and hopeless, I talk to a friend. I go and talk with a professional about my situation.

#### Relationship With the Person With SSD

Caregivers described good relationships with the persons with SSD they cared for. They provided practical daily support for the persons with SSD, such as assistance in shopping or household chores. They encouraged people with SSD to keep up their routines and stay away from substance abuse. For example, they participated in public events together without being ashamed. Caregivers discussed general topics that the person with SSD was interested in, introduced easy-to-discuss topics, and tried to get the person to think more positively. They also paid attention to whether they were showing symptoms or not. Caregivers supported the person with the illness in seeking help, even if it was a hard decision. They were involved in planning and monitoring care and attended meetings organized with mental health professionals:

I visit the city about once a week with my mother. At that time, we go banking, shops and other places.

I try to encourage her and talk about her good sides and what she is good at. The illness is not usually seen but can sometimes be, so I have decided not to be ashamed of her anywhere.

We go to public events and we have done trips together abroad.

Fortunately, at least for now I have had an opportunity to join the care meetings and my wife has wanted me to be there.

Caregivers described challenging relationships with the persons with SSD. These challenges were related to physical distance, timetables, the stage of illness, and challenges in communication. Sometimes, the physical distance between the caregiver and the person with SSD was too great for them to meet each other often. Schedules were sometimes difficult to coordinate, or there was only a short amount of time to see each other. Sometimes, in a bad stage of the illness, the person with SSD might not want to be in touch or want the caregiver to be involved if they were in hospital. The person with SSD could also be threatening toward the caregiver. Communication with the person with SSD was challenging when that person lacked insight, and caregivers had to be careful about what topics could be discussed without triggering anxiety or anger:

She lives with her partner in another city, so we are mainly in contact by phone, in the summertime we meet more.

I try to visit her weekly, but we often have scheduling conflicts, so our meetings do not always succeed. Many times, I have needed to withdraw, change the subject and let it be, because arguing with a man with lack of insight is somewhat hopeless.

Once he picked up a scythe. I looked at the exits and didn't stay long that time.

#### Experience of Health Care Services

Positive experiences related to care included the feeling of safety created by hospital care, good cooperation with health care services and support from professionals, and advantages of outpatient care. Caregivers were relieved when the person with SSD was safe in a hospital when they needed it. The cooperation with health care services was good when the person with SSD had had a long relationship with the same professionals, and caregivers were familiar with them and were able to contact them easily. Caregivers received concrete support from professionals, such as nurses and police, when they needed help getting the person with SSD into hospital care. Outpatient care supported the person, enabled them to be out of hospital, and enabled caregivers to see the person and how they were coping;

I was able to sleep a bit longer at night when she was in the hospital, and during the day I didn’t have to worry about what was waiting at home when I came back from work.

Outpatient care is also the best option for me. I can keep my husband at home and I see him every day. Likewise, I know when he goes to see his therapist and in what condition he comes home from there.

As the departments and staff became more familiar, cooperation began to work better in every way.

On the contrary, the challenges faced by caregivers included insufficient care, problems in cooperation between the caregiver and the health care service, and legislative issues. Caregivers described how physical symptoms and side effects of medicine were not taken seriously or were not appropriately dealt with, and there were challenges with substance abuse problems. Sometimes, it took long to get help for the person with SSD, and outpatient care seemed powerless. Caregivers had witnessed inappropriate behavior, such as verbal mocking and excessive use of force by the police when they had taken the person to a hospital. The person with SSD sometimes denied caregivers access to information, and the opinions or experiences of caregivers might have been ignored when professionals had contradictory views about the need for inpatient care. Furthermore, caregivers and health care professionals did not understand each other, especially if a doctor was not a native Finn. Legislation related to self-determination was a barrier to care if the patient wanted to stop their medication or treatment. Examples of the phrases are provided below:

Of outpatient care, I often stated that it was not treatment at all, but indeed neglect. If the patient does not come to the appointment once a month, no one from healthcare services goes home to see what the situation is.

The first thing that comes to mind is the physical illnesses of a mental patient. It seems to me that one doesn’t believe they exist.

They may have decades of experience about the person with illness. One would expect that cooperation would be more beneficial than detrimental.

For a long time, I tried to keep my husband to stay even in outpatient care, go to doctor’s appointments and take his medicine.

Even if the denial of information is standing, one should listen to relatives.

## Discussion

### Principal Findings

The goal of this study is 2-fold. We first evaluated the feasibility of the web-based course with caregivers who participated in a psychoeducation course by analyzing engagement and feedback on the course. Second, participants’ experiences of being a caregiver to a person with SSD were portrayed in writings produced during the web-based course. These 2 goals complemented each other by providing valuable information to both caregivers and professionals. It has already been shown that psychoeducational interventions for family caregivers of people with psychosis are effective [20]. It is therefore important to consider how web-based methods can be used to support family members, especially those who lack daily support [52]. At the same time, web-based courses could provide insights into caregivers’ daily concerns and how support from professionals could more efficiently meet the individual needs of caregivers. It has been shown that web-based methods are sometimes preferable to face-to-face methods, as some caregivers are less likely to share their inner thoughts in face-to-face counseling sessions [53,54].

Out of 34 caregivers providing informed consent, 30 caregivers started the web-based course. Engagement with the modules decreased as the course proceeded. The number of log-ins similarly decreased in a study by Rotondi et al [26]. This may be because caregivers lack the strength to continue [2,3]. Some web-based programs with different target groups have shown a similar decrease in log-ins and an increase in withdrawals during the program [55]. In our study, of the 30 caregivers, 60% (18/30) completed all the 6 modules, which is a fairly good result compared with similar studies [28].

The question of how feasible web-based psychoeducation courses are for caregivers still remains if 53% (9/17) of the participants are unsure of its usability. Therefore, we need to discuss what might have been the reason for the lower ratings in our study. First, it is possible that, considering participants’ extensive experience in caregiving, the course content did not offer new or valuable information for them, although the content of the course seemed to be interesting to the participants. Therefore, this kind of course might be more topical for people new to their role as a family caregiver. It has already been recommended that low-intensity interventions could be used as a first step of service for illnesses of milder severity before stepping up to treatments with higher intensities if needed [56].

Second, we offered opportunities for peer support via a web-based course platform. However, only a limited number of conversations between caregivers can be identified. Caregivers in our study may have been less active users of web-based discussion forums, which could have limited their willingness to have discussions via the internet. Furthermore, some participants may have been worried about the privacy issues of web-based services [57]. Third, our professional tutors offered individual support to each participant and actively contacted them if any worries could be recognized based on their writings. Caregivers might not have been used to remote support if they were familiar with face-to-face contact with professionals. On the other hand, acceptance of web-based interventions has been found to be high even if contact with professionals is limited [58]. In general, support provided by professionals seems to increase attrition [59,60]. Therefore, in practice, the preferences for psychoeducation courses and the presence of tutors or other professionals should be clearly indicated by the caregivers before the intervention. During these exceptional times in health services, we need to be able to provide web-based services effectively to those who are not able to travel to seek help or are not motivated to use web-based services.

Fourth, we may also ask whether these web-based courses should be targeted only to participants who are fully engaged and facilitated to use web-based methods or if more individual support should be offered to participants with a lack of engagement and skills. On the basis of a previous study, available technical support is important when participants lack technical skills [61]. The caregivers in our study had the possibility of asking questions via the internet [62]. Chat functions, video meetings, and video-recorded instructions could also be added to the course platform to help caregivers with any technical problems. Although these solutions may help technically oriented participants, they may not be useful to those who are less technically literate.

Fifth, the findings of this study indicate that a successful web-based psychoeducation intervention for caregivers demands that participants are supported during the course so that they will likely be engaged in the entire course. Ensuring that participants are motivated, have the necessary technology skills, and get help when needed is crucial. Engagement in the course could be supported by adding professional guidance and discussing the timing of the course and how much time should be allotted for each task of the course. On the other hand, keeping in mind refusals and dropout rates, we need to consider whom web-based psychoeducation courses should target—should only those who are familiar with web-based methods be the focus, or should everyone, even those with fewer skills in digital technology, be targeted? To answer this fundamental question, more empirical studies are still needed. In any case, researchers should be clearly aware of the challenges of tailoring the web-based method to the needs and requirements of the target group.

Caregivers reflected on their own experiences of being a family member of a person with SSD. Their writings show that caregivers have problems with their own physical and mental well-being, which is similar to earlier findings [9-11,63]. Compared with the results of the study by Pollio et al [5], who identified 355 problems caregivers faced in managing mental illness in their relatives, our study focused on positive methods and how to manage daily life. We found that, in addition to burdening experiences, caregivers had versatile methods for supporting their own well-being and they received support from people around them. This is contrary to previous studies, in which caregivers of persons with schizophrenia lacked social support [64]. This is a positive result, as social support is associated with a lower level of burden [65]. Caregivers also described their relationship with the person with SSD they cared for, including both good and challenging experiences. Caregivers were able to modify their communication, social relationships, and daily activities based on the patient’s mood and mental status. This flexible communication style would also be welcome in interactions with professionals.

Indeed, cooperation with professionals was seen as challenging, especially if they had different views of the patient’s needs in inpatient care. As found in earlier studies [66,67], caregivers had contradictory feelings when a person with SSD was admitted to a hospital. They were relieved that the person was getting help; however, they also felt that they did not receive information and that they were ignored as a resource in care. The legislation in Finland [68] determines that information contained in patient documents is confidential, and professionals are not allowed to give out information to anyone about a patient’s care without written consent from the patient. In general, confidentiality is highly respected in health care services. However, it has been found to be problematic in psychiatric services [66]. Even so, it is still possible for health care professionals to listen to the worries of caregivers, give them general information about treatment, and show empathy without breaking any confidentiality rules in patient care. Web-based psychoeducation programs could serve as a tool for understanding caregivers’ situations and offering support and knowledge in a neutral way.

### Limitations and Strengths

Our study has a number of limitations. First, because of the small sample size, the results cannot be generalized to a wider context. Second, most participants in our study were women aged around 60 years [24,25,27,28] who had been caregivers for quite a long time [28]. This means that most of them were *experienced* caregivers with long histories of being support persons for someone close to them with SSD. It may be that our target group would have benefited more from the involvement of a therapist in the support process, and therefore, our intervention might not have been properly suited to our target audience.

Third, we used the convenience sampling method, and potential participants were invited to participate in the study in the local associations. As the recruitment of this study was realized via the local family associations, caregivers in our study might have had more social support than caregivers generally have, which might have biased the results. Fourth, not all caregivers gave their feedback about the web-based course, although the option to do so was offered in quantitative and qualitative formats. It is also possible that the most critical caregivers dropped out in the early study phase, and therefore, the feedback was biased. We could have also used validated scales to collect feedback data [69], and the results could have been used to improve the course and its content in the future. Fifth, the length of each module could have been longer to allow more time to complete all the tasks. This might have decreased the dropout rate during the course. Sixth, the individual writing tasks were guided with questions, which may have focused on the content of the writings. Finally, some caregivers had technical problems at the beginning of the course, which may have discouraged their participation later in the course.

One strength of this study is that the data collection method was innovative. We used web-based methods for data collection, which have been found to be particularly useful in conducting a qualitative study with vulnerable study groups [70] and with stigmatized topics [4,71]. Typically, caregivers’ experiences of dealing with mental health disorders have often been studied using quantitative methods and questionnaires (eg, Zarit Burden Interview [72] and Camberwell Family Interview [73]). Qualitative methods using individual interviews [74], focus groups [75], and a combination of these [76] have also been used. With caregivers’ writings, we were given access to caregivers’ uninhibited thoughts and feelings based on their stories, experiences, and insight, instead of extra pressure caused by face-to-face interviews with researchers.

Although not conclusive, our descriptive, small-scale study can contribute to improving the feasibility of web-based psychosocial interventions. In addition, having regular meetings with the local family associations allowed us to develop good relationships with them and deepen our collaboration with caregivers. Flexible tailoring and support, good monitoring, and good topics should allow us to continue our studies with larger sample sizes in the future.

### Conclusions

Our study relates to the feasibility of a web-based psychoeducation course and the exploration of experiences of being a family caregiver. These focuses complement each other in that the study offers valuable information about usability and insights into the lives of caregivers. On the basis of the results, we can conclude that web-based psychoeducation may not be an optimal method for all caregivers of persons with SSD. However, it might be suitable for those who are quite new in their situation and those familiar with technological devices and social media platforms. To be successful, the use of web-based methods requires detailed planning related to content, practical arrangements, and the scheduling of the intervention. Carefully matching the target group and the web-based methods is especially key in meeting the participants’ needs and expectations. Effort should also be put into course participant selection. In addition, we can confirm that web-based courses are a usable method for obtaining rich, informative, qualitative data from writings that can increase professionals’ understanding of caregivers’ experiences and current needs. The next step is to run a larger study with sufficient power to obtain more rigorous information on the topic. We can conclude that although caregivers have versatile ways to improve their own well-being, they still need regular support and cooperation with health care professionals.

## Appendix

Multimedia Appendix 1 Number of log-ins among caregivers (N=30) for each module.

Multimedia Appendix 2 Number of caregivers who visited and completed each module.

## Abbreviations

FinFami: Finnish Central Association of Families With People With Mental Illness
ICD-10: 10th version of International Classification of Disorders
SSD: schizophrenia spectrum disorder
