# Supplementary figures and images for: Feasibility of a Web-Based Psychoeducation Course and Experiences of Caregivers Living With a Person With Schizophrenia Spectrum Disorder: Mixed Methods Study
Source: J Med Internet Res. 2021 Apr 23;23(4):e25480. doi: 10.2196/25480 (PMC8105764; doi:10.2196/25480)

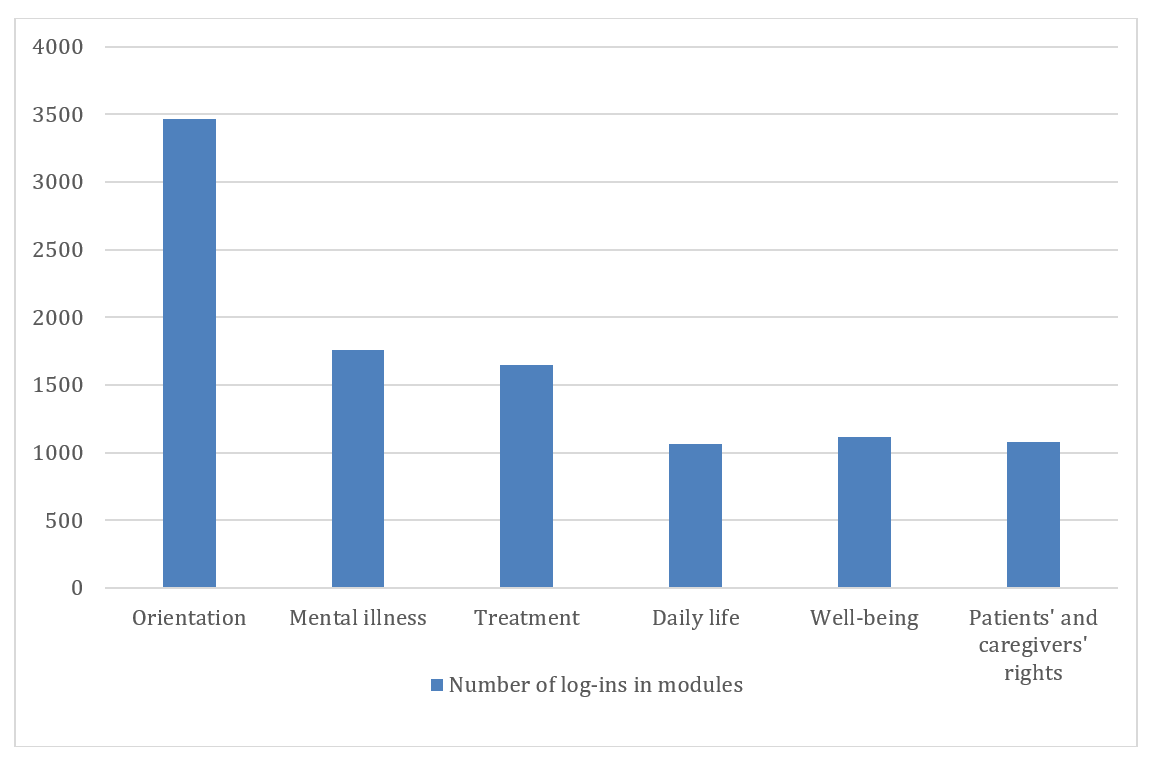

Supplement: Multimedia Appendix 1 [file jmir_v23i4e25480_app1.png]

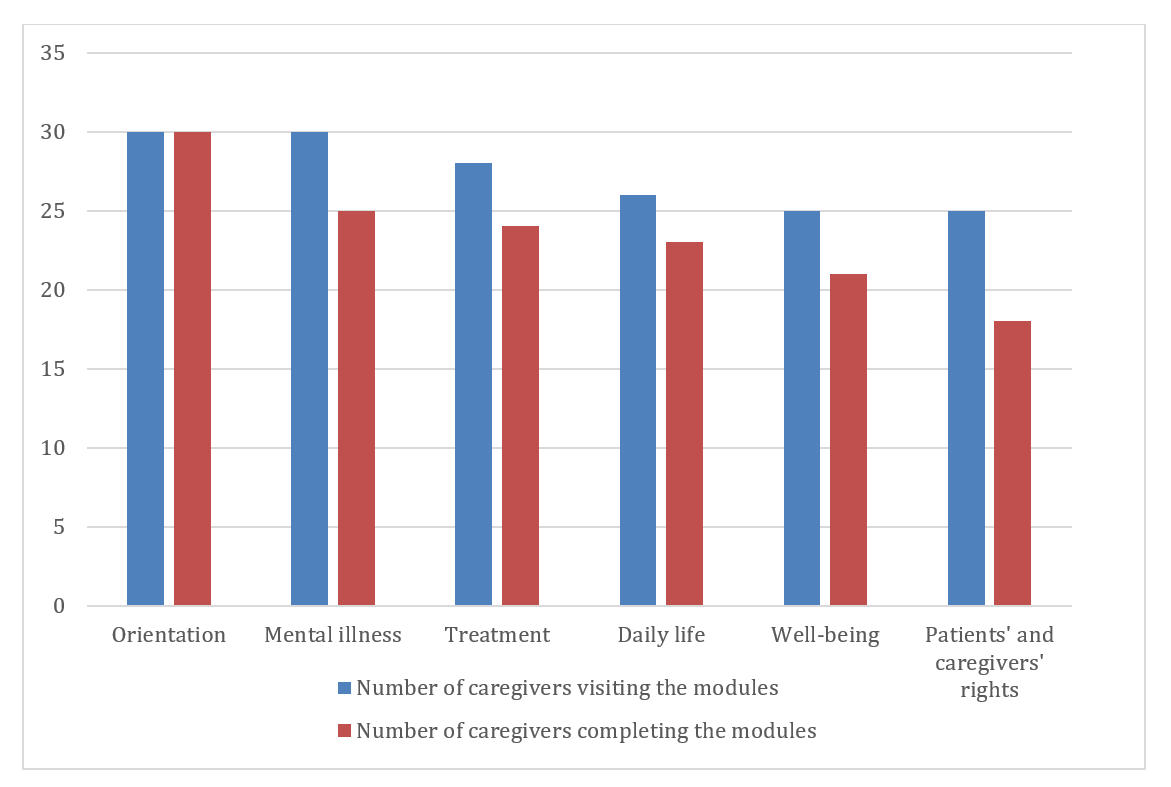

Supplement: Multimedia Appendix 2 [file jmir_v23i4e25480_app2.png]
